# Personalized Management of Erdheim–Chester Disease: The Pivotal Role of Molecular and Cross-Sectional Imaging from Staging to Targeted Therapy Monitoring

**DOI:** 10.3390/jpm16100505

**Published:** 2026-09-29

**Authors:** Stefano Piscone, Luca Pio Stoppino, Martina Matera, Benedetta Di Millo, Giorgia Schiraldi, Paola Milillo, Roberta Vinci, Luca Macarini

**Affiliations:** Department of Medical & Surgical Sciences, Section of Diagnostic Imaging, University of Foggia, Viale Luigi Pinto n. 1, 71122 Foggia, Italy; martina.matera@unifg.it (M.M.); benedetta.dimillo@unifg.it (B.D.M.); giorgiaschiraldi1997@gmail.com (G.S.); pmilillo@ospedaliriunitifoggia.it (P.M.); roberta.vinci@unifg.it (R.V.); luca.macarini@unifg.it (L.M.)

**Keywords:** Erdheim–Chester disease, histiocytosis, cross-sectional imaging, coated aorta, hairy kidney, cardiovascular involvement

## Abstract

Erdheim–Chester disease (ECD) is a rare, clonal non-Langerhans cell histiocytic neoplasm driven by activating mutations in the mitogen-activated protein kinase (MAPK) pathway, most notably the BRAF-V600E mutation. The discovery of these molecular alterations has shifted the therapeutic paradigm from non-specific immunosuppression to personalized targeted therapies, such as BRAF and MEK inhibitors, dramatically improving patient outcomes. Given its protean and insidious systemic manifestations, imaging plays an indispensable role in the personalized diagnostic and therapeutic pathway of ECD. Metadiaphyseal symmetrical osteosclerosis represents the skeletal hallmark, while extraskeletal involvement typically affects the cardiovascular, retroperitoneal, pulmonary, and central nervous systems, yielding pathognomonic imaging signatures such as the “coated aorta” and “hairy kidney.” Advanced cross-sectional techniques—including CT, MRI, and organ-specific mapping protocols—are crucial for baseline phenotype characterization, risk stratification, and guiding biopsies toward hypermetabolic sites for biomarker detection. Furthermore, molecular imaging with ^18^F-FDG PET/CT has emerged as the cornerstone for baseline systemic staging and quantitative metabolic response monitoring via PERCIST criteria, enabling early assessment of targeted treatment efficacy long before morphological changes occur. This review illustrates the multimodality imaging spectrum of ECD based on our institutional experience, emphasizing how precision imaging integrates with molecular profiling to guide personalized patient management.

## 1. Introduction

Erdheim–Chester disease (ECD) is a rare, aggressive non-Langerhans cell histiocytic neoplasm characterized by systemic infiltration of CD68-positive, CD1a-negative histiocytes, with a wide spectrum of clinical manifestations [1]. This immunophenotypic profile distinguishes ECD from Langerhans cell histiocytosis (LCH).

Initially described as “lipoid granulomatosis” by William Chester and Jakob Erdheim in 1930 [2], ECD was originally considered an inflammatory histiocytosis. However, the discovery of activating mutations in the mitogen-activated protein kinase (MAPK) signaling pathway established ECD as a histiocytic neoplasm, although inflammation continues to contribute substantially to organ damage [3].

Accordingly, ECD was included in the “histiocytic dendritic cell neoplasms” category in the 2016 World Health Organization classification of hematopoietic tumors [4].

### 1.1. Epidemiology

The estimated incidence is fewer than one case per million people per year. Although the precise global prevalence remains uncertain because of the lack of comprehensive national registries, more than 1500 cases have been documented worldwide since 1930 [5].

The disease predominantly affects adults, with a mean age at diagnosis of approximately 55 years. Most patients are between 50 and 70 years of age, although cases have been reported in patients ranging from 7 to 84 years old [6].

There is a marked male predominance. Studies indicate that around 60–70% of cases occur in men, with an estimated male-to-female ratio of 1:5.

No specific regional or ethnic predisposition has been identified. ECD has been reported in North America, Europe, Asia, and other regions worldwide. However, more cases have been reported in countries with advanced healthcare systems, where disease awareness and access to diagnostic techniques are greater. Pediatric ECD is rare (<20 reported cases) and usually spares the heart; affected children may initially present with central nervous system juvenile xanthogranuloma [7,8].

Over the past fifteen years, the number of ECD diagnoses has increased substantially. This trend does not necessarily reflect an increase in disease prevalence, but rather greater awareness within the scientific community and improved clinical recognition [9].

### 1.2. Pathophysiology & Molecular Landscape

ECD is characterized by tissue infiltration by lipid-laden (foamy) histiocytes and Touton giant cells which produce proinflammatory cytokines and chemokines and harbor somatic mutations in genes that activate the MAPK/ERK and the PI3K/AKT signaling pathways [10].

Among the most relevant alterations is BRAF V600E, an aminoacid substitution that constitutively activates the BRAF protein. It is identified in approximately 50–70% of cases and leads to constitutive activation of the MAPK signaling cascade, promoting histiocyte proliferation and survival [1]. The mutation was first identified in 2010 in a landmark study of patients with Langherans cell histiocytosis (LCH) and, two years later was shown to be recurrent and to occur at a similar frequency in histiocytes from ECD lesions [11,12]. BRAF V600E has also been detected in CD34+ hematopoietic cells, supporting a bone marrow origin of the disease.

Another common alteration involves MAP2K1, and results in constitutive, RAF-independent activation of the MEK1 protein kinase and downstream MAPK signalling [13,14]. As a downstream effector within the RAS-RAF-MEK-ERK cascade, activated MEK1 phosphorylates ERK1/2, resulting in persistent stimulation of histiocyte proliferation. Thus, both upstream mutations and constitutive activation of the MEK-ERK pathway contribute to the phenotypic basis of extensive systemic involvement [1,15].

In particular, the deposition of lipid-laden histiocytes in various organs and the expression of the p16Ink4a marker by ECD cells induce an inflammatory state that may lead to fibrotic deposits in multiple tissues and organs [3]. This process, together with xanthelasma-like lesions and various endocrine deficiencies, contributes to the major clinical manifestations of the disease. The classic histopathological features are foamy histiocytes surrounded by fibrotic tissue and lymphocytes [16].

This explains why, although interferon alfa therapy has traditionally been used to treat Erdheim–Chester disease, targeted therapies, such as BRAF and MEK inhibitors, have revolutionized the therapeutic landscape and significantly improved patient outcomes [5,17].

### 1.3. Clinical Presentation and Role of Imaging

The clinical presentation is highly heterogeneous and often insidious. Manifestations include systemic symptoms, such as fever of unknown origin, weight loss, severe fatigue and night sweats, which are present at onset in over a third of patients as well as organ-specific symptoms ranging from dull, persistent lower-limb bone pain to diabetes insipidus, exophthalmos, cerebellar disorders, renal failure due to obstructive uropathy, and cardiac decompensation secondary to tamponade or myocardial ischemia [5,18].

In this context, diagnostic imaging plays a key role in directing diagnostic suspicion when characteristic findings are present; guiding biopsy toward extraskeletal sites with greater metabolic activity or accessibility; mapping systemic disease, particularly in areas with the greatest prognostic impact; and quantitatively monitoring therapeutic response in patients receiving BRAF inhibitors (e.g., vemurafenib, dabrafenib) or MEK inhibitors (e.g., cobimetinib) [1] (Table 1).

### 1.4. Aim

Although historically considered exceedingly rare and frequently under-recognized, the number of diagnosed cases has increased substantially over the past 15 years, largely owing to improved awareness, advances in molecular pathology, and better recognition of characteristic imaging findings.

The aim of this pictorial review is to illustrate the spectrum of imaging manifestations of Erdheim–Chester disease across multiple organ systems, based on our institutional experience, and to highlight the key radiological patterns that may facilitate an earlier diagnosis and improve familiarity with this uncommon but clinically significant disease.

## 2. Distribution

Erdheim–Chester disease (ECD) has a highly heterogeneous pattern of anatomical involvement, reflecting the ability of pathological histiocytes to infiltrate connective, perivascular, and stromal tissues in almost all organ systems.

Despite this variability, lesion distribution is not random: the disease shows a marked predilection for specific anatomical sites, including the skeletal system, retroperitoneum, cardiovascular system, central nervous system and adventitial layers of the great vessels), the combination of which defines the characteristic radiological and clinical phenotypes (Table 2).

### 2.1. Skeleton

Skeletal involvement represents the hallmark of Erdheim–Chester disease and is observed in approximately 80–95% of patients [19]. It typically manifests as bilateral and symmetrical osteosclerosis involving the metaphyseal and diaphyseal regions of the long bones (Figure 1), with relative sparing of the epiphyses and axial skeleton, unlike LCH [20]. The lower limbs are most affected, particularly the femur, tibia, and fibula, while involvement of the ulna, radius, and homerus is less frequent [21].

Skeletal disease is symptomatic in nearly 38% of cases and usually presents with mild to moderate bone pain, most localised around the knees and ankles. 

In some patients, pain may require treatment with nonsteroidal anti-inflammatory drugs or, less commonly, opioid analgesics. Because of its highly characteristic bilateral and symmetric distribution, long-bone involvement is considered one of the most distinctive radiological features of ECD and often provides the first diagnostic clue. 

Conventional radiographs demonstrate bilateral and symmetric cortical and medullary osteosclerosis localized to the metaphysis and diaphysis of the long bones. Narrowing or obliteration of the medullary canal may be observed, together with endosteal thickening and diffuse periosteal apposition (periostitis).

High-resolution computed tomography (HRCT) demonstrates markedly increased trabecular bone attenuation (with replacement by dense sclerotic bone, >800 HU), obliteration of the normal medullary architecture, solid endosteal sclerosis, cortical remodeling, and irregularity of the periosteal surface.

On magnetic resonance imaging (MRI), T1-weighted imaging reveals complete replacement of the normal high-fat marrow (marrow fat replacement) with pathological tissue showing a symmetrical meta-diaphyseal distribution. T2-weighted/STIR imaging reveals variable signal intensity, typically heterogeneously hyperintense on fat-suppressed sequences (STIR/T2 Fat-Sat), reflecting active medullary oedema, histiocytic infiltration and acute inflammation. In the late/chronic stages, hypointense signal may predominate on T2-weighted sequences as dense fibrosis and sclerosis develop.

Diffusion-weighted imaging (DWI) and apparent diffusion coefficient (ADC) maps may demonstrate restricted diffusion within areas of active cellular infiltration, reflecting the high cellular density of lipid-laden histiocytes.

Post-contrast sequences show hypervascularisation of the stroma and the presence of active fibro-inflammatory tissue.

Nuclear medicine plays a central role in the assessment of skeletal disease. Whole-body bone scintigraphy (WBS) using 99mTc-methylene diphosphonate (99mTc-MDP) has historically been the standard modality for documenting hallmark skeletal lesions. WBS classically demonstrates intense, strictly bilateral and symmetric tracer uptake involving the metaphysis and diaphysis of the long bones, particularly in the lower extremities (distal femur and proximal and distal tibia) (Figure 2). This avid cortical and periosteal uptake produces the characteristic “double-strip sign” (or symmetric peripatellar and supra-malleolar hyper-concentration), with sharply demarcated boundaries and relative sparing of the epiphyses and axial skeleton. This scintigraphy pattern is present in more than 95% of patients and provides near-complete specificity in distinguishing ECD from other histiocytic disorders such as Langerhans cell histiocytosis, which typically presents with focal, asymmetric osteolytic lesions involving the calvarium, pelvis, and axial skeleton. In parallel, molecular imaging with ^18^F-FDG PET/CT complements bone scintigraphy by detecting metabolically active osteo-medullary disease.

PET/CT is the modality of choice for quantitative monitoring of the metabolic response during targeted therapies (PERCIST criteria) [22,23].

### 2.2. Bone Marrow

ECD is currently recognised as an inflammatory myeloid neoplasm, and bone marrow involvement may contribute to haematological abnormalities, particularly microcytic anaemia, which may occasionally represent one of the earliest clinical manifestations of the disease. However, alternative causes of anaemia, including nutritional deficiencies, hereditary disorders, acute or chronic blood loss, and anaemia of chronic disease, should first be carefully excluded.

The presence of bone marrow involvement also raises the possibility of associated myeloproliferative neoplasms or myelodysplastic syndromes, which should be considered during diagnostic work-up. In selected cases, particularly when haematological abnormalities are unexplained or persistent, bone marrow biopsy may be useful to support diagnosis and exclude concomitant haematological disorders [16].

PET/CT is the modality of choice for baseline systemic assessment of the bone marrow [22].

### 2.3. Cardiovascular System

Advances in cross-sectional imaging have significantly improved recognition of cardiovascular involvement in Erdheim–Chester disease. Cardiovascular involvement is identified in 50–60% of patients with ECD, making it one of the most common and clinically critical extra skeletal manifestations.

ECD typically affects the adventitia of the aortic tree, as well as the pericardium, right atrioventricular (AV) groove, interatrial septum, and coronary arteries, particularly the right coronary artery (RCA).

Computed tomography (CT) may demonstrate circumferential peri-aortic infiltration, producing the characteristic “coated aorta” appearance (Figure 3). This fibro-inflammatory process may involve both the thoracic and abdominal aorta and extend to its major branches [24]. Despite its often-extensive appearance, peri-aortic involvement is typically clinically silent and is not usually associated with aneurysmal dilatation, dissection, or rupture.

Coronary artery involvement is identified in approximately one-quarter of cases and may lead to clinically significant complications, including luminal narrowing and myocardial infarction.

Pericardial involvement is also common and encompasses a spectrum ranging from pericardial thickening and pericarditis to effusion and, in severe cases, cardiac tamponade, which may be life-threatening [25].

Cardiac manifestations may also include infiltration of the atrioventricular sulcus and myocardial infiltrates. These lesions may show late gadolinium enhancement on cardiac MRI and can be associated with atypical infarct-like patterns that do not conform to conventional vascular territories.

When coronary involvement is present, stenosis may affect the coronary ostia and the right coronary artery. Although atherosclerotic coronary artery disease is not specifically associated with cardiac ECD, suspicion of ECD should be raised when coronary insufficiency results from extrinsic compression related to atrial or atrioventricular sulcal infiltration [26].

The differential diagnosis includes disorders that may mimic the cardiovascular manifestations of ECD. For example, large-vessel vasculitis (LVV) may present with similar findings. An ECD case with subclavian and carotid artery stenosis and aortitis has been reported, potentially mimicking LVV; however, LVV generally lacks the characteristic systemic extravascular manifestations of ECD [27].

Another well-recognized manifestation is the presence of a right atrial pseudotumor, which can be detected in a considerable subset of patients, particularly on dedicated cardiac magnetic resonance imaging (MRI).

Cardiac involvement includes pericardial effusions, ischemic heart disease and cardiac failure [28]. Thickening of the right atrium creates pseudo-tumours that are best detected by cardiac MRI (Figure 4 and Figure 5). Circumferential aortic wall thickening occurs in two-thirds of patients [8].

Literature affirms that right AV groove infiltration with right coronary artery encasement is the most common ECD cardiac localization. In addition, it can lead to myocardial edema and variable degrees of myocardial fibrosis with coronary distribution. The localization and the transmural distribution of myocardial LGE (but also alteration of native T1 values in mapping-sequences) can suggest the presence of a cardiac ischemic pattern due to the extrinsic compression or direct infiltration of coronary artery walls by histiocytes, that can affect coronary hemodynamic and determinate extracellular matrix remodeling processes [29].

In summary, key radiological findings are: coated aorta (a continuous, circumferential periaortic sheath of fibro-inflammatory soft tissue enveloping the thoracic and abdominal aorta), right atrial pseudotumour and atrioventricular groove encasement (a pseudotumour-like soft-tissue infiltrative mass within the right atrioventricular groove that encases the right coronary artery (RCA) and causes thickening of the right atrial wall), extrinsic coronary sheath (circumferential compression of soft tissues at the level of the coronary ostia and proximal arterial segments, without primary formation of endovascular atheromatous plaques). In these cases, imaging methods of choice are CT, which is indicated for vascular assessment, as well as for evaluating cardiac morphology and the pericardium; MRI, which is important for tissue characterization, mapping sequences and late gadolinium enhancement; and ^18^F-FDG PET/CT, which demonstrates intense uptake along the walls of the aorta and marked focal uptake in cardiac pseudotumours.

Although cardiovascular manifestations are frequently dominant in institutional series due to the high diagnostic yield of cardiac MRI and contrast-enhanced CT, their balanced appraisal alongside other systemic sites is crucial. The detection of a ‘coated aorta,’ right atrial pseudotumors, and atrioventricular groove infiltration must always be evaluated in parallel with multiorgan staging rather than as isolated findings, ensuring an integrated assessment of both morphological adventitial disease and functional myocardial consequences.

### 2.4. Retroperitoneum

Retroperitoneal and renal involvement is observed in 50–60% of patients with ECD [30]. Although renal involvement is frequently asymptomatic, symptomatic patients may present with flank or dull lower-back pain, dysuria, hypertension due to renal artery compression, and acute or chronic renal failure secondary to bilateral ureteral obstruction [31].

Perirenal infiltration is generally bilateral and symmetric, typically involving Gerota’s fascia, the renal capsule, perirenal fat, and the renal sinus, with extension along the proximal two-thirds of the ureters and into the periaortic retroperitoneal soft tissues.

Retroperitoneal fibrosis is observed in approximately one-third of patients with ECD and may be complicated by bilateral hydronephrosis and proximal ureteral obstruction (in almost 50% of cases), occasionally requiring ureteral stenting [32]. In ECD, the fibrotic process typically circumferentially encases the aorta, whereas in idiopathic retroperitoneal fibrosis, the posterior aortic wall is usually spared.

Importantly, this sheathing process involves not only the aorta and renal capsule but also frequently extends to the proximal ureters, representing the main mechanism of ureteral obstruction in affected patients. Abdominal CT commonly demonstrates perirenal fat infiltration, producing the characteristic “hairy kidney” appearance (Figure 6), which is observed in a substantial proportion of patients [19].

On computed tomography (CT), the perirenal space demonstrates bilateral, symmetric infiltrative soft-tissue attenuation replacing the normal perirenal fat. Dense, irregular, spiculated or filamentous projections radiate from the renal capsule into the retroperitoneum, producing the characteristic “hairy kidney” appearance.

The underlying renal parenchyma is typically intact and not displaced, although it is slightly compressed (the ‘book-page kidney’ phenomenon). The fibrotic mass envelops the renal hilum, the renal arteries and veins, without evidence of vascular thrombosis or endoluminal invasion.

The periaortic retroperitoneal soft-tissue sheath extends anteriorly, laterally and posteriorly until it completely encloses the abdominal aorta and the inferior vena cava (IVC). It extends progressively downwards to involve the proximal and middle segments of both ureters, causing extrinsic luminal narrowing and secondary hydronephrosis.

On magnetic resonance imaging (MRI), T1-weighted images show homogeneous hypointense or isointense signal relative to skeletal muscle, replacing the normally hyperintense perirenal fat. T2-weighted images demonstrate low-to-intermediate signal intensity. Active inflammatory disease may show patchy T2 hyperintensity within the perirenal infiltrate, whereas chronic fibrotic disease typically demonstrates uniform low T2 signal.

On ^18^F-FDG PET/CT, the perirenal and periaortic regions may demonstrate circumferential, bilateral, symmetric, and intense FDG uptake, with elevated baseline SUVmax values (SUVmax = 5.0–11.0), reflecting metabolically active histiocytic infiltration. PET/CT is therefore useful for distinguishing active retroperitoneal inflammation from inactive fibrotic scar tissue and for monitoring response to targeted BRAF/MEK inhibition.

Therefore, the pathognomonic/key radiological findings are: the ‘hairy kidney’ sign (bilateral, symmetrical, spiculated/filamentous infiltration of the soft tissues of the perirenal fat and Gerota’s fascia), circumferential periaortic encasement (360° encasement of the retroperitoneal abdominal aorta and inferior vena cava, extending into the perirenal space), proximal/mid-ureteral encasement (extrinsic encasement of the upper two-thirds of the ureters, resulting in bilateral hydronephrosis) [30].

In a small minority of patients, a solid renal mass resembling renal cell carcinoma has been reported [16].

The differential diagnosis of retroperitoneal involvement includes IgG4-related disease (IgG4-RD), a fibro-inflammatory disorder that shares several manifestations with ECD, including pleural, pericardial, pulmonary, pituitary, retroperitoneal, and large-vessel involvement. In addition to histological differences, IgG4-RD generally lacks the characteristic long-bone involvement of ECD, whereas ECD typically lacks lacrimal and salivary gland involvement, pancreato-cholangitis, and tubulointerstitial nephritis. MRI may also help distinguish their patterns of fibrosis: ECD characteristically shows circumferential periaortic thickening and proximal ureteral involvement, whereas IgG4-RD more commonly demonstrates anterolateral periaortic fibrosis and distal ureteral involvement [27].

By harmonizing the cross-sectional evaluation of the retroperitoneum, the classic ‘hairy kidney’ sign and ureteral encasement should be systematically correlated with ^18^F-FDG PET/CT metabolic activity. This integration prevents over-reliance on pure morphology, allowing clinicians to accurately differentiate active fibro-inflammatory retroperitoneal progression from burnt-out, acellular scar tissue following targeted therapy.

### 2.5. Central Nervous System

Central nervous system (CNS) involvement occurs in up to 50% of patients with ECD and predominantly affects the hypothalamic–pituitary axis, cerebellum and brainstem (particularly the dentate nuclei and middle cerebellar peduncles), pachymeninges (dural folds, falx, and tentorium), and retro-orbital fat (bilateral retrobulbar, extraconal, and intraconal spaces) [33,34] (Figure 7). CNS involvement is clinically important and has been identified as an independent adverse prognostic factor for overall survival.

Clinical manifestations are heterogeneous and largely dictated by the sites of involvement. 

Diabetes insipidus is the most frequent presentation, while other manifestations include seizures, pontocerebellar dysfunction, and a broad spectrum of neuroendocrine abnormalities, notably panhypopituitarism [35].

Less commonly, patients may present with neuropsychiatric symptoms or progressive cognitive decline.

The posterior fossa is frequently involved (Figure 8), typically demonstrating multifocal FLAIR hyperintensities involving the dentate nuclei and brainstem, often associated with variable contrast enhancement and, in some cases, regional atrophy [31,36].

A recent MRI study of 58 patients with ECD reported cortical, brainstem, cerebellar, and pituitary lesions in 46.6%, 29.3%, 25.9%, and 17.2% of patients, respectively. Notably, the BRAF V600E variant was significantly associated with cerebellar and bilateral brainstem involvement [37].

Supratentorial involvement is less common but may also be encountered. Dural-based enhancing lesions are observed in a subset of patients and may occasionally display a stellate morphology [38].

Sellar imaging may reveal enlargement of the pituitary gland and/or infundibulum, sometimes accompanied by signal abnormalities, including loss of the normal T1 hyperintensity of the posterior pituitary. Orbital/optic nerve involvement is relatively frequent and is characterized by infiltrative soft-tissue masses, often associated with exophthalmos [39] (Figure 9).

Bilateral, progressive, painless exophthalmos/proptosis, diplopia, restricted ocular motility, and optic nerve compression leading to visual field deficits may occur. Concomitant osteosclerosis of the maxillofacial skeleton may also be present (Figure 10).

Recent evidence confirms that orbital involvement is predominantly bilateral, with intraconal masses representing a common imaging pattern. Proptosis and extraocular muscle restriction are among the most frequent ophthalmic manifestations [40]. Targeted therapy with BRAF inhibitors may also result in substantial improvement of orbital manifestations, particularly proptosis, in patients harboring BRAF-V600E mutations [41].

Spinal involvement is less common and demonstrates variable imaging features, including multifocal intramedullary T2-hyperintense enhancing lesions [42] and extramedullary masses that may compress or infiltrate the spinal cord. Patchy brainstem enhancement, a frequent finding, may mimic entities such as Chronic Lymphocytic Inflammation with Pontine Perivascular Enhancement Responsive to Steroids (CLIPPERS) or CNS lymphoma; however, it typically appears more confluent than CLIPPERS and lacks the characteristic steroid responsiveness of these conditions [43]. 

In some cases, ECD may mimic IgG4-related disease (IgG4-RD) because of retro-orbital inflammation [27,38,44]. 

On ^18^F-FDG PET/CT, intense hypermetabolism may be observed in retro-orbital masses, the pituitary stalk, and areas of pachymeningeal thickening.

The assessment of brainstem and cerebellar lesions on PET may be influenced by the physiological uptake of the tracer by the cortex; for longitudinal metabolic monitoring, basic PET/MRI fusion protocols or dedicated cerebral PET protocols are recommended.

Pathognomonic/key radiological findings are, ultimately: bilateral retrobulbar masses (symmetrical infiltration of the retro-orbital adipose tissue surrounding the optic nerves and muscle bellies without bone erosion), loss of the T1 signal intensity of the posterior pituitary (thickening of the infundibulum associated with loss of the normal T1 signal intensity of the posterior pituitary in patients with diabetes insipidus), symmetrical T2 hyperintensity of the dentate nuclei and middle cerebellar peduncles (MCP) (bilateral and symmetrical hyperintensity on FLAIR/T2 sequences localised in the dentate nuclei and middle cerebellar peduncles (MCP), coated dura mater/pachymeningeal thickening (laminiform masses, with enhancement, of dural origin mimicking meningiomas along the falx and the tentorium).

Central nervous system involvement represents a critical prognostic sanctuary that requires a balanced multi-compartmental imaging approach. Coordinating dedicated sellar, posterior fossa (dentate nuclei/middle cerebellar peduncles), and pachymeningeal protocols ensures that subtle neuro-endocrine structures (such as the pituitary stalk) are evaluated with the same rigor applied to macro-structural dural-based lesions.

### 2.6. Lungs

Pulmonary involvement is identified on thoracic CT in approximately 30–50% of patients with ECD. Imaging may demonstrate both pleural and pulmonary parenchymal infiltration, often with an interstitial lung disease-like pattern. The most common finding is smooth interlobular septal thickening; less frequent manifestations include micronodules, ground-glass opacities, parenchymal consolidation, interlobar fissural thickening, and occasional cystic changes, the latter being more typical of Langerhans cell histiocytosis [45].

Pleural involvement, frequently right sided, may be symptomatic and can pose diagnostic challenges. Although these radiological patterns are relatively common, persistent respiratory symptoms such as respiratory failure are uncommon, and pulmonary function tests are often only minimally impaired [46]. Nevertheless, careful assessment of thoracic involvement remains essential because it has been identified as an independent adverse prognostic factor in multivariable analyses [47].

Pathognomonic/key radiological findings are: symmetrical thickening of the interlobular septa (diffuse, smooth to nodular, bilateral thickening of the interlobular septa, mimicking cardiogenic pulmonary oedema in the absence of heart failure), peribronchovascular and subpleural lining (axial interstitial thickening extending continuously along the bronchovascular bundles), nodular pleural thickening with effusion (concomitant pleural thickening, smooth or nodular, with bilateral free-flowing fluid effusion in the absence of primary pleural neoplasia).

In contrast to the prominent cardiovascular and retroperitoneal phenotypes, pulmonary involvement in Erdheim–Chester disease—though identified in a substantial subset of patients—frequently presents with subtle or non-specific radiological patterns. A balanced diagnostic approach must carefully weigh smooth interlobular septal thickening, peribronchovascular interstitium involvement, and pleural changes against cardiogenic or infectious mimics, acknowledging that pulmonary manifestations, while less visually dominant, carry significant prognostic weight (Figure 11).

### 2.7. Skin

Xanthelasma represents the most common cutaneous manifestation of ECD, typically involving the eyelids and periorbital regions [18]. Compared with conventional xanthelasma palpebrarum, lesions associated with ECD more frequently involve the reticular dermis and are characterized histologically by a higher density of multinucleated and Touton giant cells, with relatively limited fibrosis. Additional cutaneous findings may include nonspecific patches or papulonodular lesions, most distributed over the lower extremities, back, and trunk. Involvement of the vulva and clitoris is considerably less frequent and is less characteristic than in Langerhans cell histiocytosis.

## 3. Differential Diagnosis

Distinguishing ECD from other fibro-inflammatory and autoimmune mimics remains a major diagnostic challenge. Large-vessel vasculitis (LVV) may present with carotid and subclavian stenoses and aortitis that closely mimic the “coated aorta” appearance of ECD; however, LVV typically lacks widespread multiorgan extraskeletal manifestations such as symmetric long-bone osteosclerosis and “hairy kidneys.” Similarly, IgG4-related disease (IgG4-RD) substantially overlaps with ECD, including retroperitoneal fibrosis, pleural and pericardial involvement, and pituitary stalk infiltration. Important distinguishing features include the absence of characteristic long-bone involvement in IgG4-RD and the relative absence of salivary/lacrimal gland involvement and sclerosing cholangitis in ECD. Cross-sectional imaging may further aid differentiation: ECD characteristically demonstrates circumferential (360°) periaortic sheathing and proximal ureteral involvement, whereas IgG4-RD more commonly manifests as focal anterolateral periaortic fibrosis and distal ureteral involvement. Finally, CNS manifestations of ECD, such as T2 hyperintensity of the dentate nuclei and middle cerebellar peduncles, should be differentiated from Chronic Lymphocytic Inflammation with Pontine Perivascular Enhancement Responsive to Steroids (CLIPPERS); compared with CLIPPERS, ECD lesions are typically more confluent and do not show the characteristic response to corticosteroid therapy.

## 4. Systemic Multimodality Imaging Strategy

Because Erdheim–Chester disease (ECD) is a multisystemic histiocytosis with protean clinical manifestations multimodality imaging is indispensable at baseline to establish the diagnosis, map the full extent of organ involvement, identify accessible biopsy targets, and monitor treatment response over time (Figure 12).

For baseline skeletal screening, conventional radiography and whole-body bone scintigraphy with 99mTC-MDP serve as the primary diagnostic gateway. The identification of bilateral and symmetrical metadiaphyseal hyperfixation forming the classic ‘double-strip sign’ provides an immediate, high-specificity diagnostic fingerprint even in patients presenting with nonspecific systemic manifestations or asymptomatic bone involvement. While ^18^F-FDG PET/CT has superseded bone scanning for longitudinal metabolic response assessment, baseline WBS remains an invaluable, cost-effective confirmation tool that rapidly guides the clinical suspicion toward ECD before initiating advanced systemic cross-sectional imaging [48,49,50].

^18^F-FDG PET/CT (from the base of the skull to mid-thigh/whole body) is the most effective basic screening tool for the whole body. It enables the determination of the overall disease burden, the mapping of metabolic activity and the precise identification of hypermetabolic sites for percutaneous biopsy (particularly when skeletal lesions are densely sclerotic and yield poor-quality histological tissue) (Figure 13).

In a registry study of 50 patients with ECD, Kirchner et al. demonstrated the complementary value of metabolic and anatomic imaging: 20% of disease sites were detected by ^18^F-FDG PET alone, whereas an additional 23% were identified by brain MRI alone. Moreover, among lesions measurable according to formal response criteria, 76% were evaluable by modified PERCIST but not by RECIST, highlighting the added value of metabolic imaging for disease assessment and clinical trial eligibility [51].

Contrast-enhanced CT of the chest, abdomen, and pelvis is essential for high-spatial-resolution assessment of vascular adventitial infiltration (“coated aorta”) [51], retroperitoneal infiltration (“hairy kidney”), and interlobular septal thickening on high-resolution CT [52,53,54].

Organ-specific MRI of the brain/orbits and heart provides dedicated baseline assessment of high-risk sites, including the hypothalamic–pituitary axis, dentate nuclei, orbits, right AV sulcus, and myocardium, regardless of clinical symptoms [55].

For longitudinal treatment monitoring, ^18^F-FDG PET/CT is the preferred functional imaging modality because metabolic changes may be detected even when persistent fibrosis limits interpretation of morphologic response. Modified PERCIST criteria have been applied in prospective therapeutic trials and ECD imaging cohorts; however, no ECD-specific metabolic response criteria or SUV thresholds have yet undergone formal prospective validation. Therefore, metabolic response should be interpreted together with clinical findings and organ-specific CT or MRI. Evidence supporting imaging strategies in ECD remains heterogeneous. Although prospective therapeutic studies and prospective patient registries are available, much of the imaging literature consists of retrospective single-centre cohorts, small case series, and expert consensus. In a registry-based study of 50 patients, ^18^F-FDG PET/CT identified 20% of disease sites not detected by conventional CT/MRI, whereas brain MRI identified an additional 23% of sites, emphasizing the complementary rather than interchangeable role of metabolic and organ-specific anatomic imaging. Among lesions measurable by formal response criteria, 76% were evaluable by modified PERCIST alone. These findings support multimodality assessment but should not be interpreted as validation of universal ECD-specific PET thresholds [56].

The 2014 ECD consensus guidelines established the first structured imaging framework for systemic evaluation, recommending chest–abdomen–pelvis CT, whole-body ^18^F-FDG PET including the brain and distal extremities, contrast-enhanced brain MRI, and cardiac MRI at baseline. The 2020 molecular-era consensus retained this multimodality imaging backbone but expanded its role within a precision-medicine framework. Lesional biopsy is now strongly emphasized not only for histopathologic confirmation but also for BRAF V600E and broader MAPK–ERK pathway characterization, while full-body FDG-PET/CT is integrated with organ-specific imaging for biopsy targeting and treatment-response assessment. After initiation of a new therapy, full-body FDG-PET/CT and imaging of involved organs are recommended at approximately 2–6-month intervals, with subsequent imaging frequency individualized once a stable best response has been documented. Thus, the major evolution from 2014 to 2020 is not merely an expansion of imaging modalities, but the transition from diagnostic staging toward molecularly integrated treatment selection and longitudinal response monitoring.

## 5. Treatment Strategies

Targeted therapies have transformed the treatment of ECD by directly inhibiting molecular alterations that drive the disease [5].

BRAF inhibitors, such as vemurafenib and dabrafenib, are first-line treatment options for patients with the BRAF V600E mutation (present in approximately 50–60% of cases). Vemurafenib was approved by the FDA in 2017 specifically for ECD and has demonstrated high response rates. A systematic review confirmed the efficacy of vemurafenib in patients with BRAF^V600-mutated ECD, demonstrating high and durable response rates, although treatment-related adverse events and the limited size of available clinical studies remain important considerations [57].

Dabrafenib represents an alternative BRAF inhibitor and may have a more favorable cutaneous tolerability profile [58].

Recent long-term real-world data confirmed the high efficacy of BRAF inhibitor monotherapy, with an overall response rate of 85%; however, treatment discontinuation was frequent, mainly due to adverse events, highlighting the challenges associated with prolonged therapy [59].

MEK inhibitors, such as cobimetinib and trametinib, are used in patients with specific molecular alterations such as MAP2K1 mutations, as well as in patients with BRAF-wild-type disease or intolerance to BRAF inhibitors. These agents inhibit the MAPK signaling pathway downstream of RAF [60].

Interferon-α (IFN-α), including pegylated IFN-α, was historically used as first-line therapy and remains relevant in selected settings. Its immunomodulatory and antiproliferative effects may suppress pathological histiocytes. Interferon therapy has been associated with improved long-term survival, although clinically significant adverse effects, including fatigue and neurological symptoms, may occur [61].

Because ECD is characterized by marked inflammation, anti-cytokine therapies have also been investigated. Anakinra, an IL-1 receptor antagonist, has been shown to reduce pain and inflammatory symptoms, although its effects on CNS and cardiac involvement appear limited. Tocilizumab, an IL-6 receptor antagonist, has shown clinical activity in small patient series [62].

Other treatment options include corticosteroids, such as prednisone, which may be used for rapid symptom control during acute inflammatory phases or in combination with other agents. Immunosuppressive therapies such as sirolimus, an mTOR inhibitor, may be considered in selected patients who do not respond to interferon or who lack a BRAF mutation [63].

These therapeutic advances have substantially improved prognosis, with the reported 3-year mortality rate decreasing from 60% to 20% in recent years. Current research includes clinical trials such as the COBRAH trial of cobimetinib [64] and studies aimed at determining whether and when treatment can be safely discontinued without increasing the risk of relapse, with the broader goal of further personalizing patient management.

## 6. Limitations

Published diagnostic-performance data are limited and should be interpreted in the context of small and predominantly retrospective cohorts. In one retrospective nuclear-medicine series evaluating 49 bone scintigraphy examinations, skeletal scintigraphy showed a sensitivity of 100%, specificity of 97%, positive predictive value of 90%, and negative predictive value of 100% for characteristic ECD skeletal involvement. Comparable universally applicable sensitivity and specificity values are not available for CT, MRI, or ^18^F-FDG PET/CT across the full spectrum of ECD because lesion detectability varies substantially according to the involved organ and because histopathologic verification of every imaging abnormality is rarely feasible. Accordingly, published performance estimates should not be interpreted as general diagnostic thresholds. Additional methodological limitations of the available literature include referral and selection bias toward patients with multisystem or clinically severe disease at tertiary referral centres, spectrum bias related to the marked phenotypic heterogeneity of ECD, and verification bias because histological confirmation is generally obtained from only one or a limited number of disease sites rather than from every imaging abnormality. Retrospective pictorial series are also susceptible to image-selection bias, with florid and visually characteristic manifestations being preferentially represented. These limitations restrict the generalizability of reported diagnostic-performance estimates and reinforce the need for prospective, multicentre imaging studies using standardized acquisition and response-assessment protocols.

## 7. Conclusions

ECD is a multiorgan inflammatory histiocytic neoplasm that most frequently involves the skeletal system and produces highly characteristic osteosclerosis around the knees, with increased radiotracer uptake on nuclear medicine studies. Other typical imaging findings include perinephric infiltration (“hairy kidneys”), periaortic soft-tissue infiltration (“coated aorta”), and a right atrial pseudotumor. Radiological findings should be interpreted in conjunction with clinical and histopathological features to establish the diagnosis. In addition to supporting diagnosis, imaging is essential for defining disease extent, identifying targets for tissue sampling, assessing treatment response, and contributing to prognostic evaluation.

## Figures and Tables

**Figure 1:**
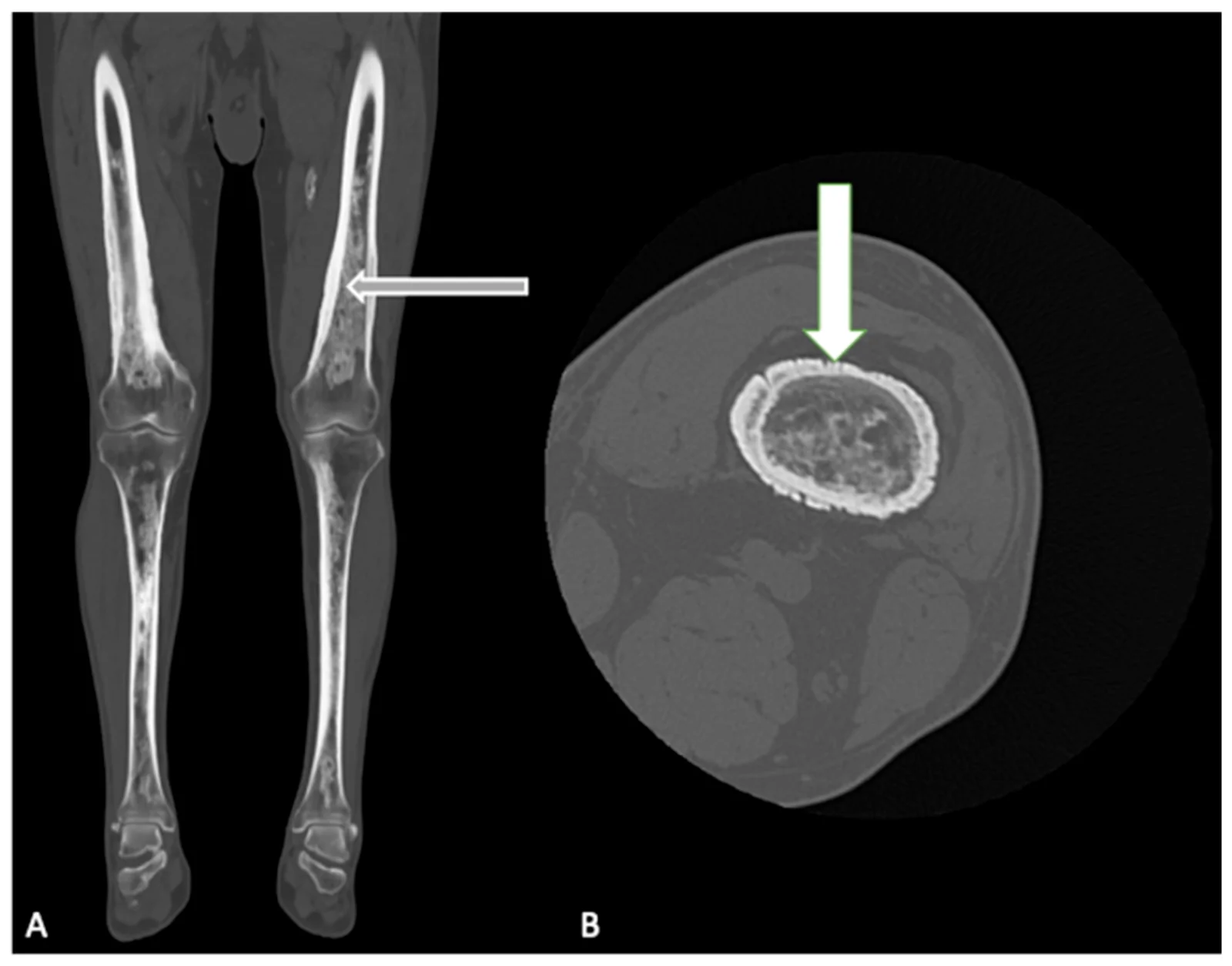
Unenhanced CT scans in coronal (**A**) and axial (**B**) planes in a patient diagnosed with ECD demonstrate pathological involvement of the appendicular skeleton, with periosteal sclerosis (white arrow) and endomedullary sclerosis (grey arrow) in the bilateral femorotibial metaphyseal–diaphyseal regions.

**Figure 2:**
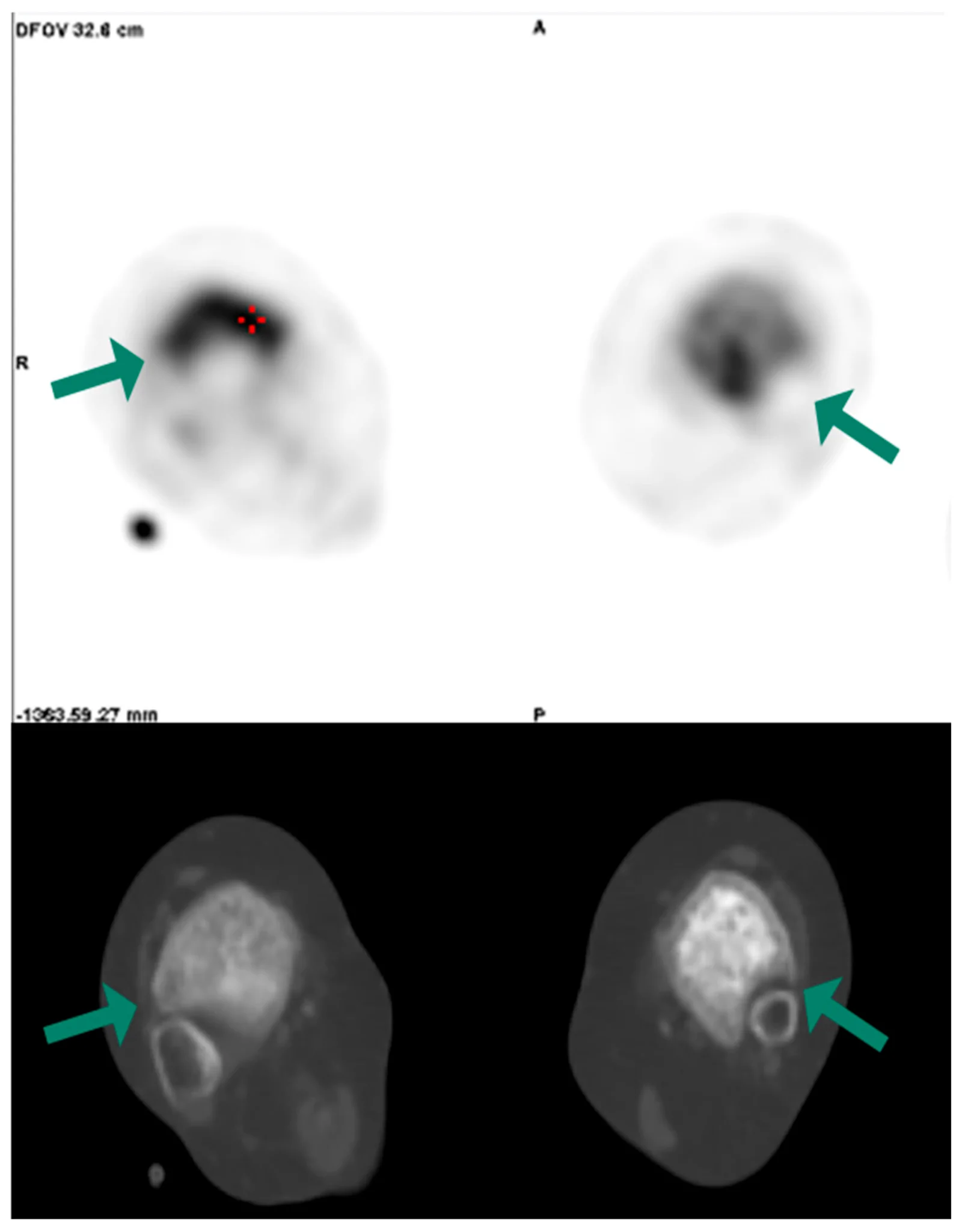
^18^F-FDG PET CT shows extensive “bone-in-bone” bone-forming remodeling in the distal third of both tibiae and in the foot bones, with similar uptake intensity (SUV max 4.2).

**Figure 3:**
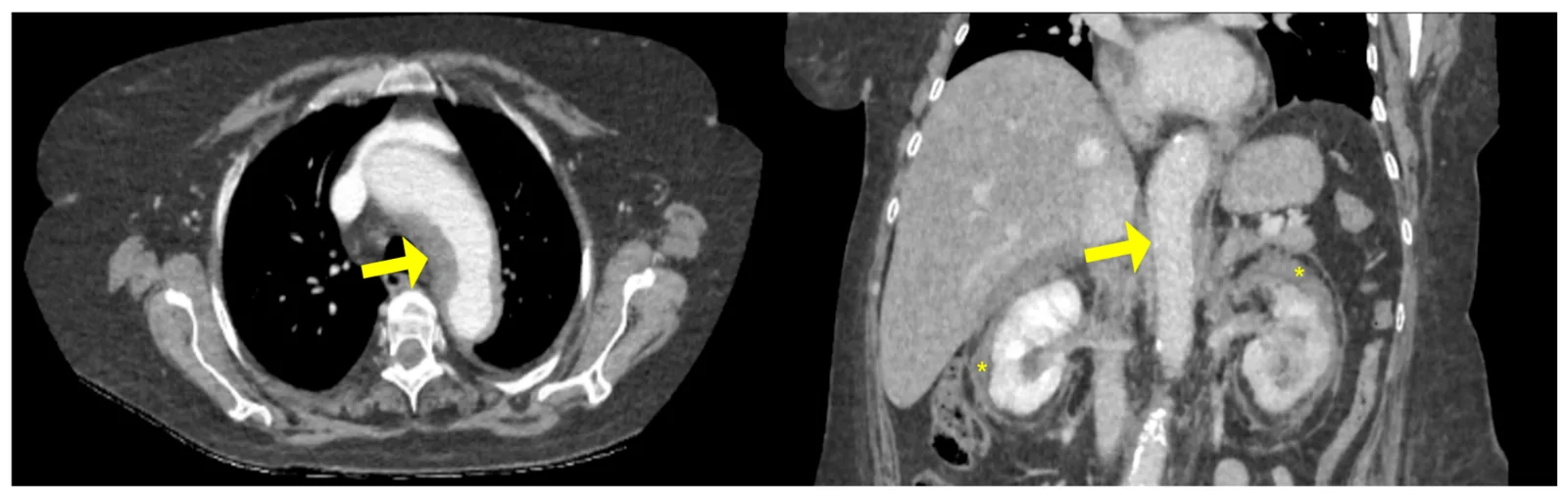
Arterial Phase Enhanced CT. The yellow arrow indicates a hypodense tissue that surrounds the thoracic aorta, extending to the suprarenal segment of the abdominal aorta. This finding is referred to in the literature as the “coated aorta sign”. The asterisk indicates symmetrical perirenal fibrotic tissue with a “hairy kidney” appearance.

**Figure 4:**
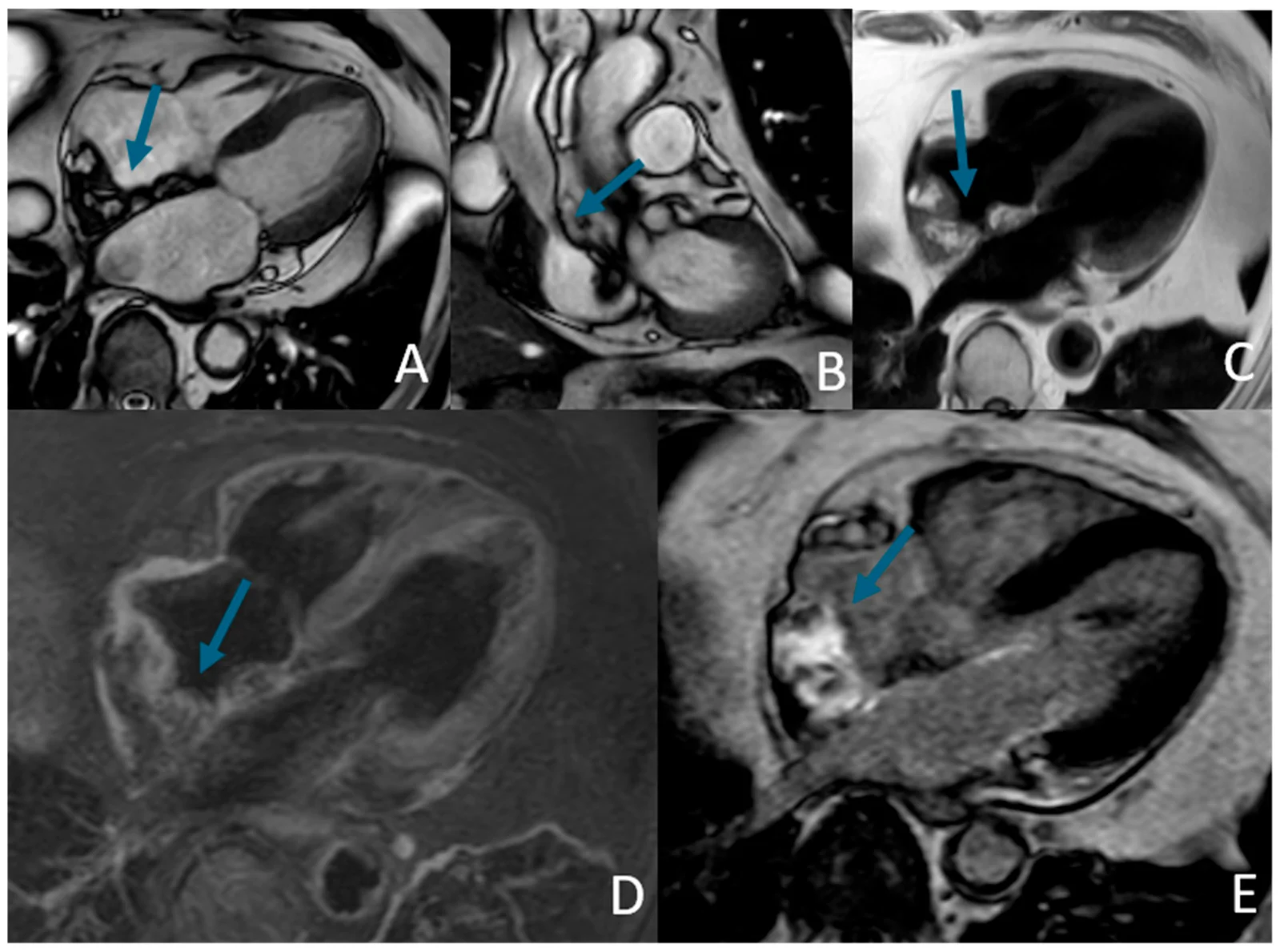
Axial sBTFE-BH (**A**), coronal s-BTFE (**B**), axial T1-BB (Black Blood) (**C**), axial T2-STIR (**D**), axial PSIR (Phase Sensitive Inversion Recovery) per Late Gadolinium Enhancement (**E**) in a patient diagnosed with Erdheim–Chester disease (ECD) showing the presence of lobulated solid tissue arising from the atrial wall and protruding into the cavity, extending along the posterior wall up to the interatrial septum (IAS). This tissue exhibits predominantly fibrotic signal characteristics (late contrast enhancement; blue arrow (**E**)), with focal areas of adipose signal (hyper-intense in (**A**–**C**) and hypo-intense in (**D**)). It encases the ostium of the superior vena cava resulting in significant luminal narrowing (blue arrow (**D**)).

**Figure 5:**
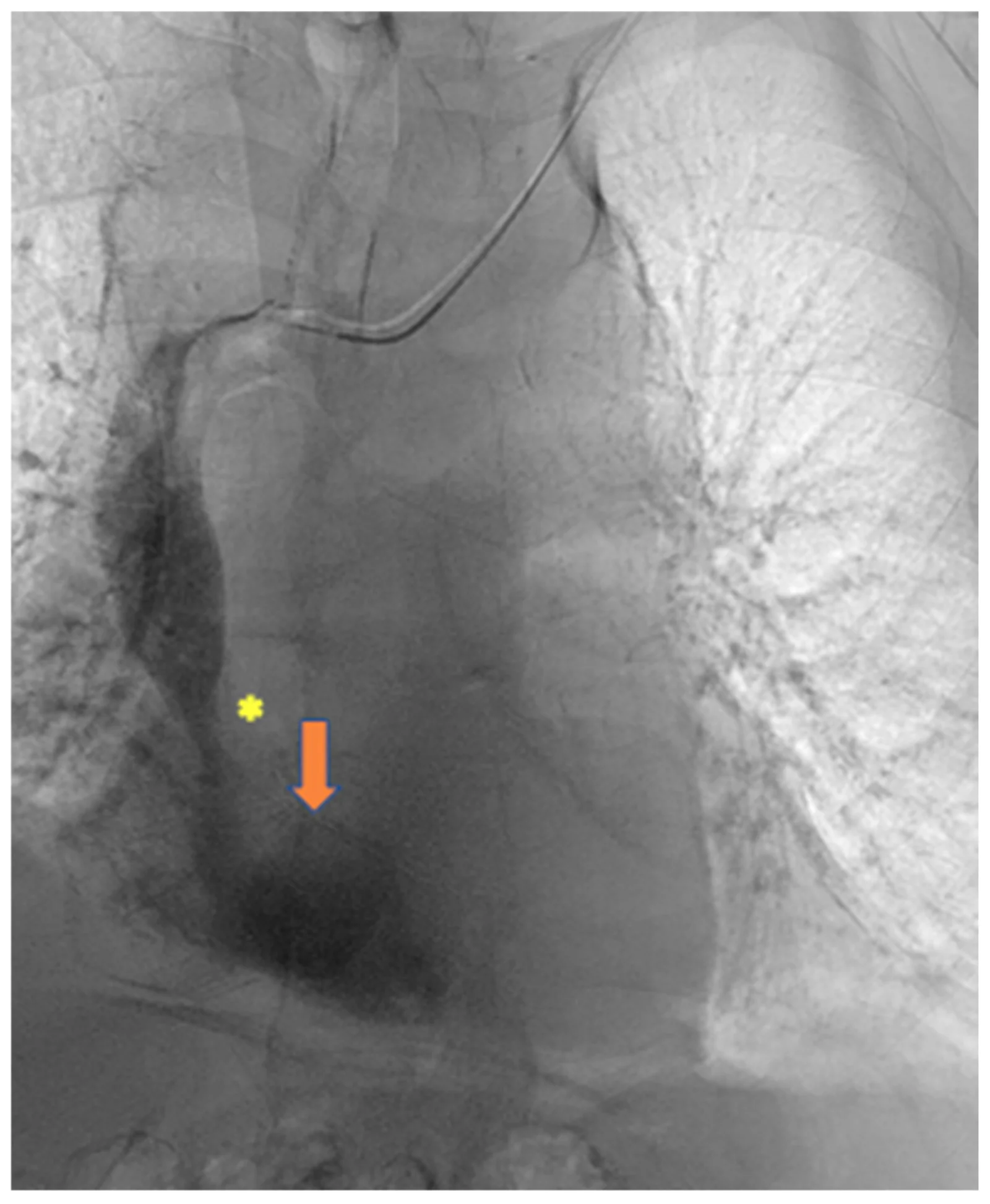
Left upper limb phlebography and superior cavography were performed in a patient with a diagnosis of Erdheim–Chester disease (ECD) via venous access through the left cephalic vein at the left forearm, with selective catheterization of the left brachiocephalic (innominate) vein. In the coronal projection, a >70% luminal narrowing of the superior vena cava at its atrial junction is demonstrated (yellow asterisk), attributable to extrinsic compression from an atrial lesion, without significant delay in contrast outflow into the right atrium (orange arrow).

**Figure 6:**
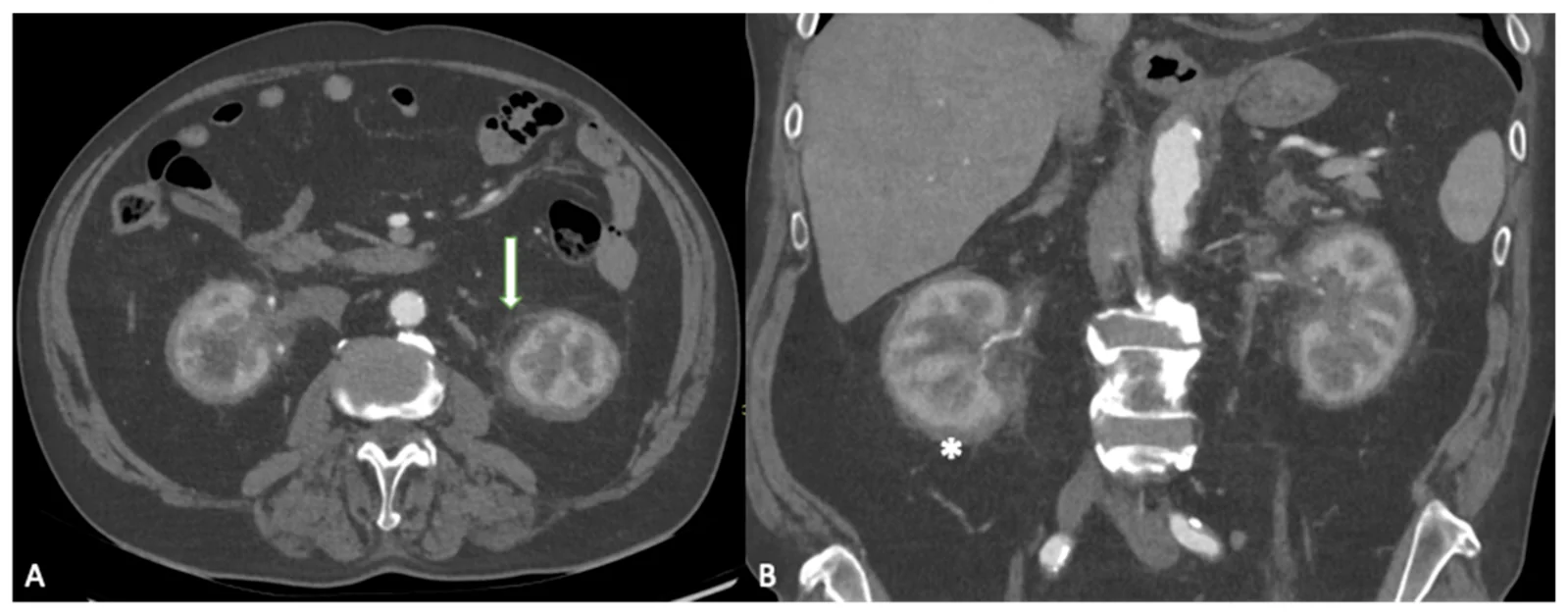
Contrast-enhanced CT scans acquired in the venous phase, displayed in the axial plane (**A**) and coronal plane (**B**), in a patient affected by Erdheim–Chester disease, demonstrate bilateral, symmetric perirenal fibrotic tissue forming a rind-like encasement of the kidneys, with irregular spiculated extensions into the surrounding fat (“hairy kidney” sign).

**Figure 7:**
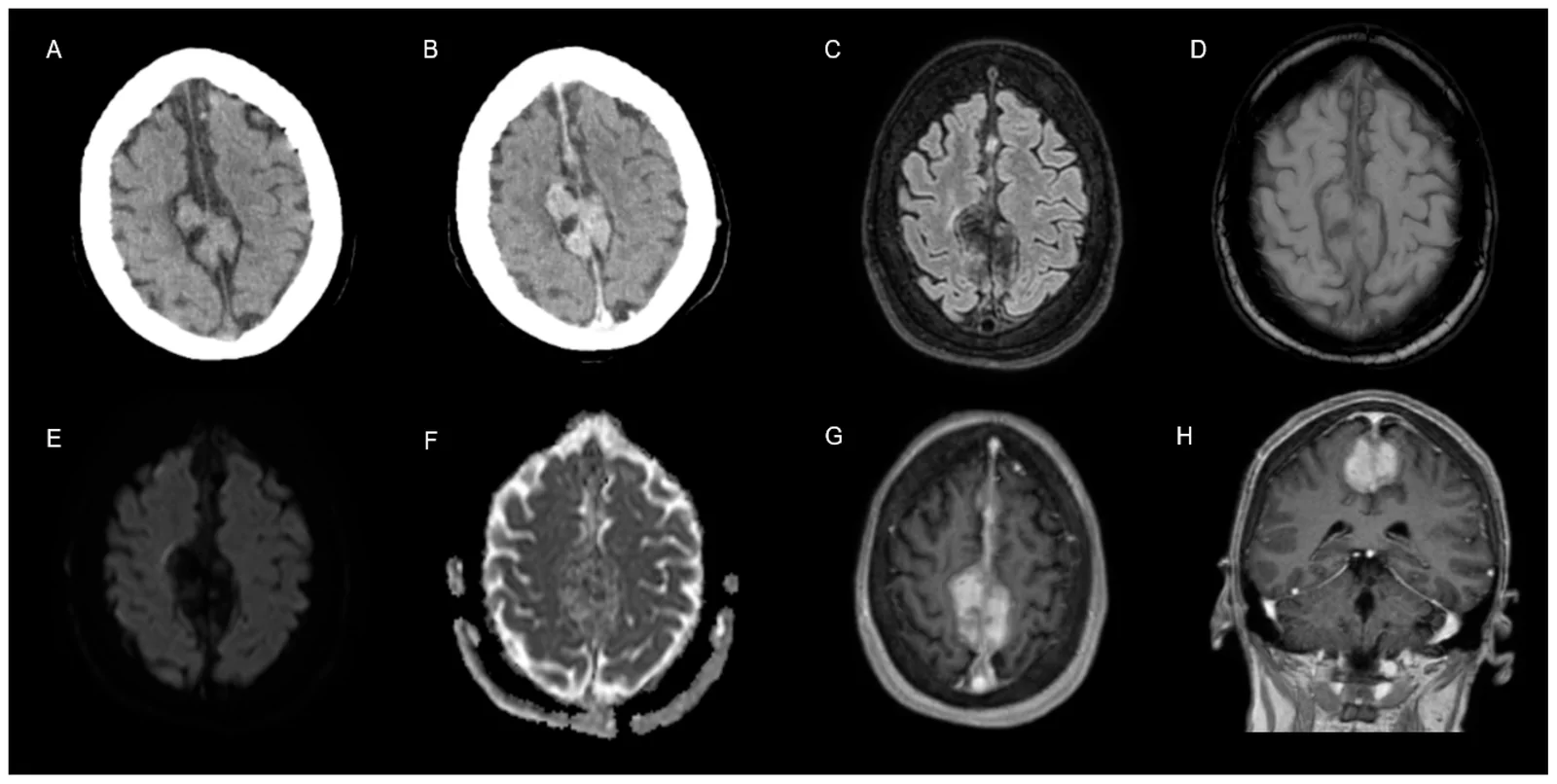
(**A**) Non-enhanced CT, (**B**) Contrast Enhancement CT, (**C**) FLAIR-T2w, (**D**) TSE-T1, (**E**) DWI, (**F**) ADC, (**G**,**H**) T1w contrast enhancement in axial and coronal view. An extra-axial, parafalcial expansive lesion with heterogeneous contrast enhancement, associated with diffuse pachymeningeal thickening.

**Figure 8:**
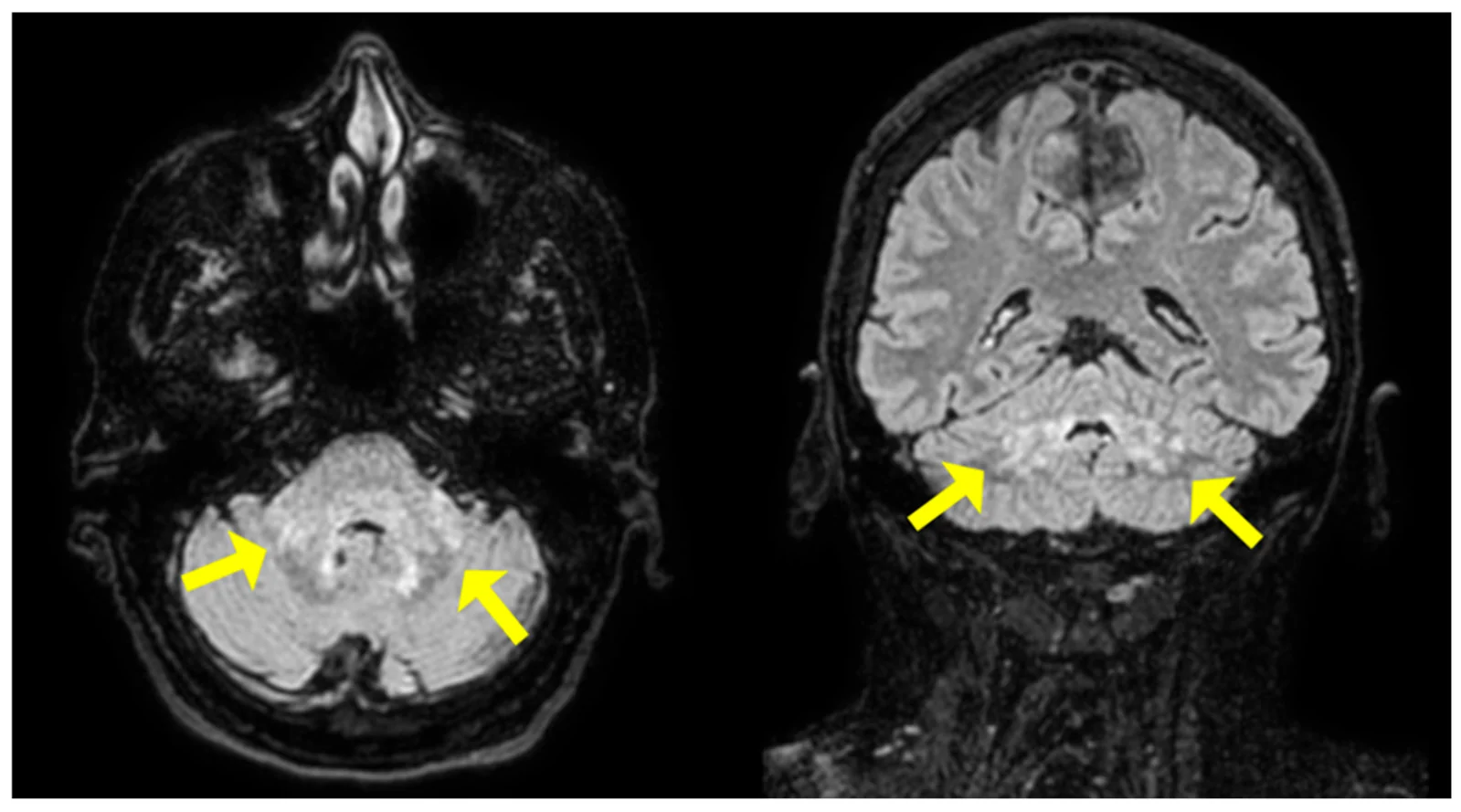
FLAIR-T2W images enlightened focal areas of signal abnormality within the cerebellar peduncles.

**Figure 9:**
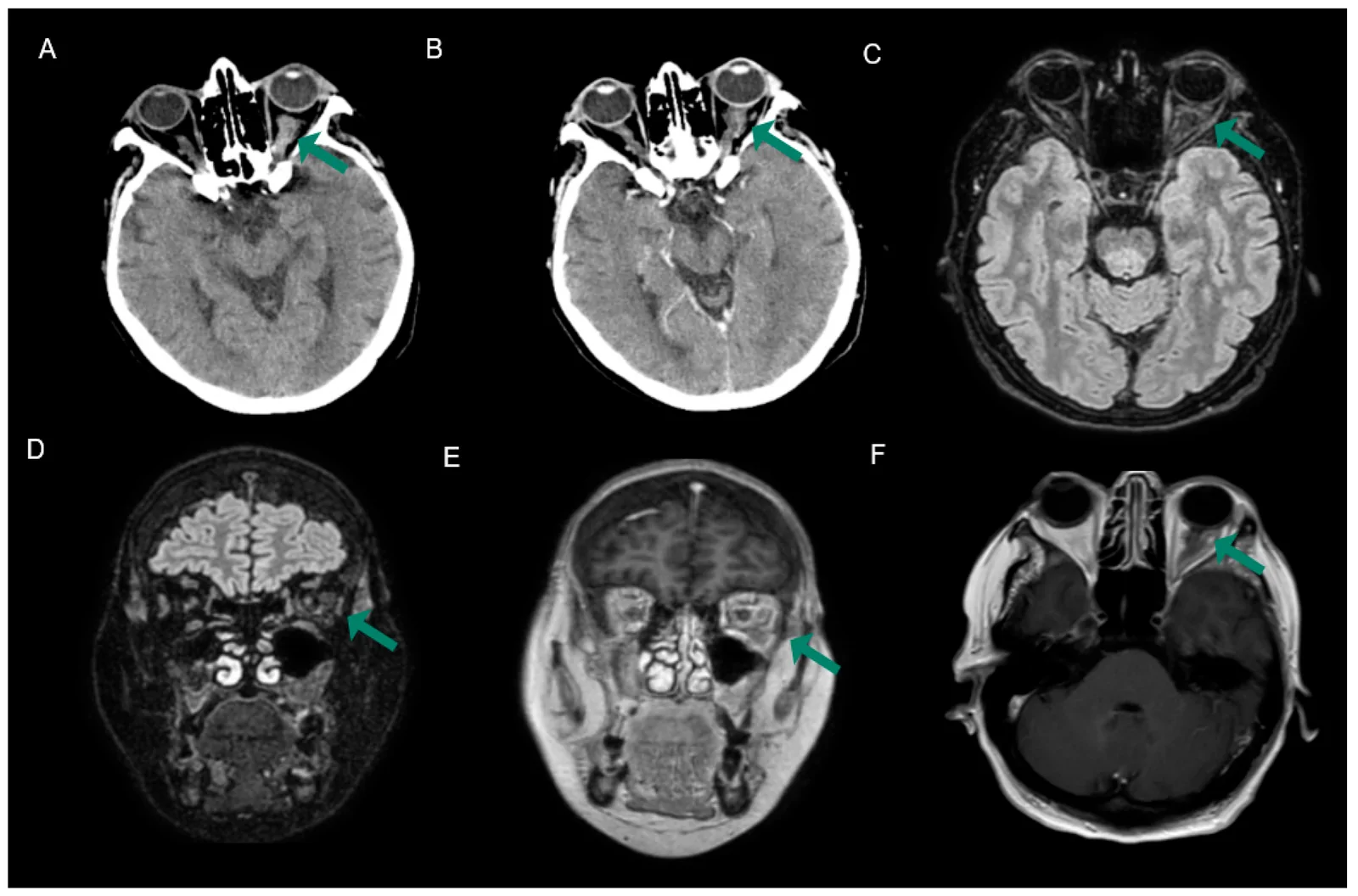
(**A**) Non-enhanced CT, (**B**) Contrast Enhancement CT, (**C**,**D**) FLAIR-T2w, (**E**,**F**) T1w contrast enhancement. The images show an expansive intracranial lesion with mild contrast enhancement along the lateral aspect of the optic nerve.

**Figure 10:**
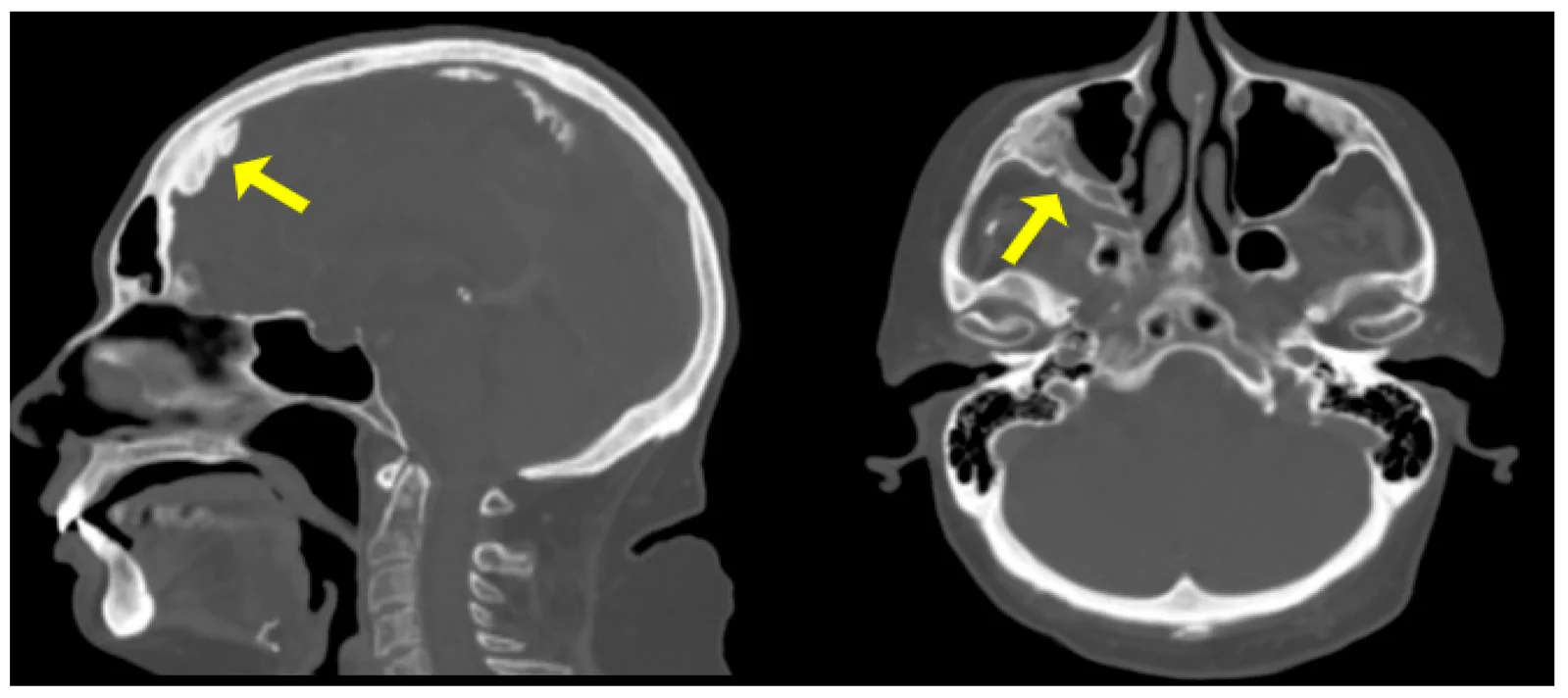
Unenhanced CT with sagittal and axial reconstructions demonstrates thickening of the craniofacial bones, including exostoses of the frontal bone and the posterolateral wall of the right maxillary sinus.

**Figure 11:**
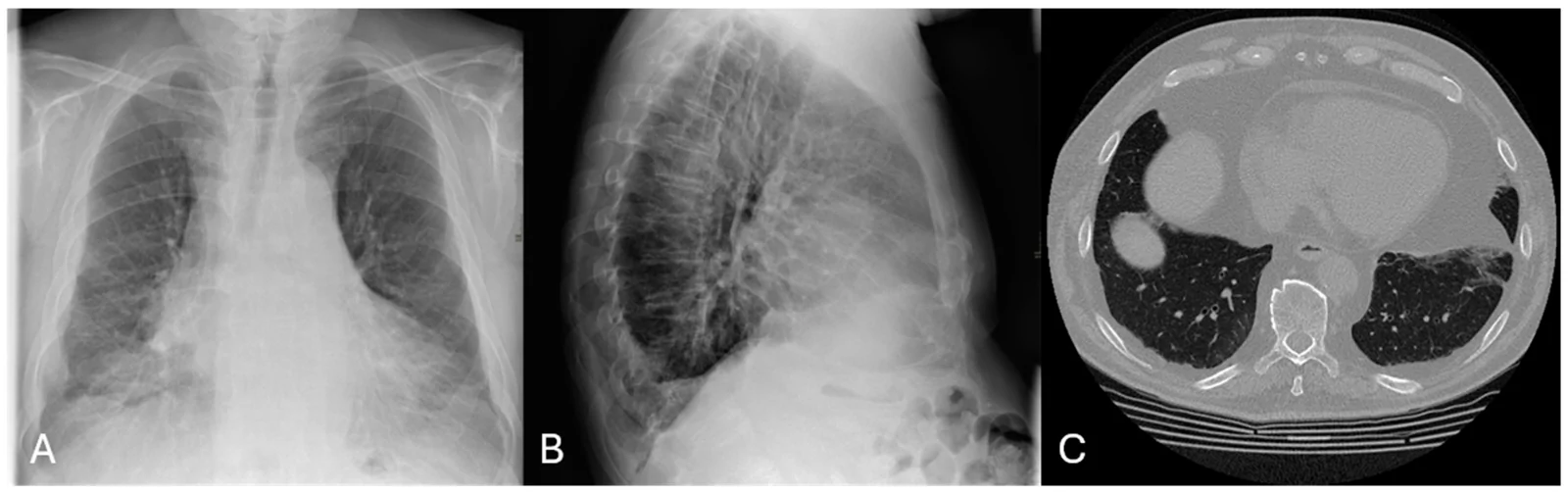
Pleuropulmonary involvement in Erdheim–Chester disease. Frontal (**A**) and lateral (**B**) chest radiographs demonstrate bilateral bibasal reticular-interstitial opacities and pleural abnormalities. Axial chest CT in lung window (**C**) confirms bilateral pleural thickening with small pleural effusions and associated subpleural/interstitial reticular abnormalities, predominantly involving the lung bases.

**Figure 12:**
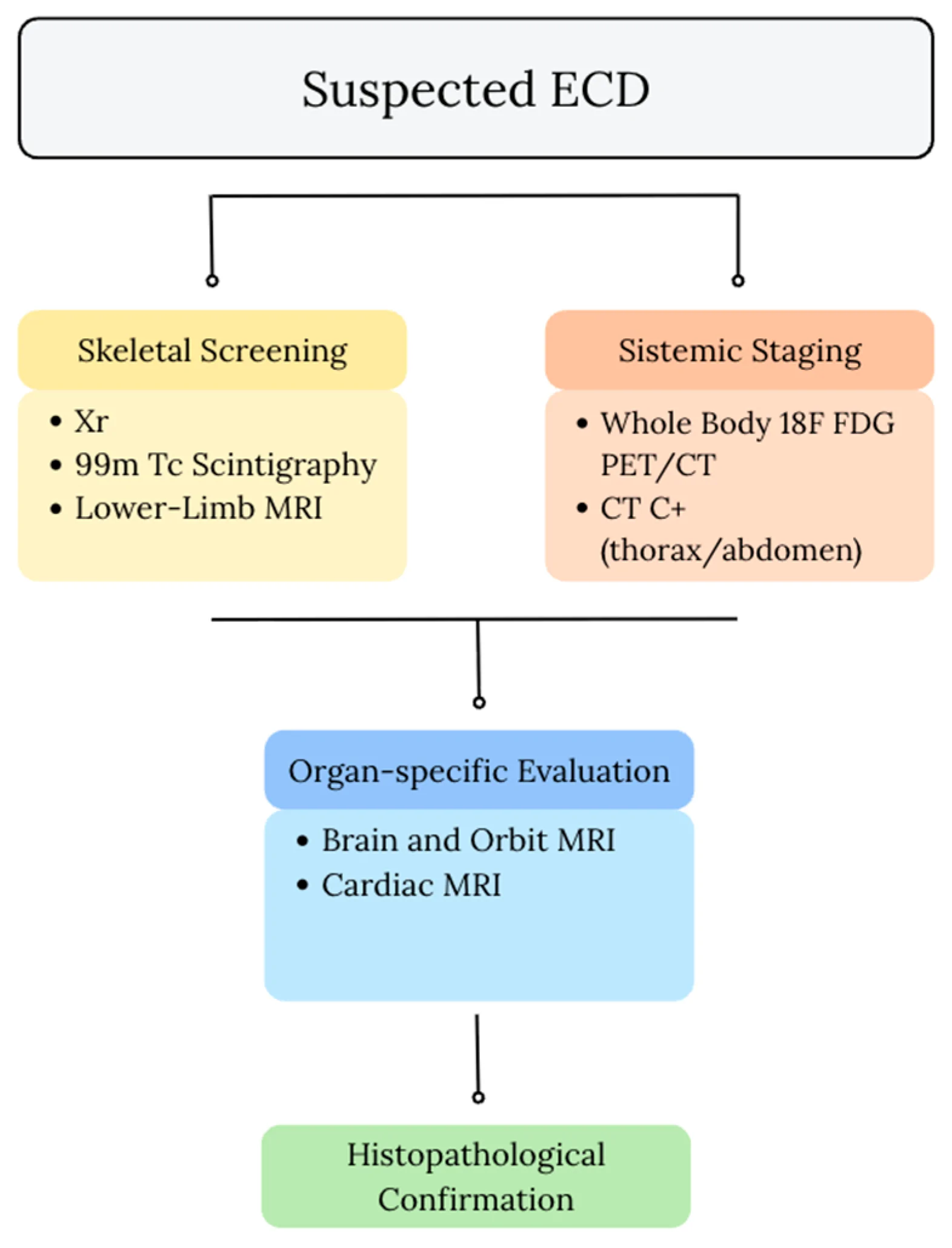
Multimodality diagnostic imaging algorithm for suspected Erdheim–Chester disease (ECD). The proposed workflow integrates skeletal screening, systemic ^18^F-FDG PET/CT and cross-sectional staging, organ-specific brain/orbital and cardiac MRI, and histopathological confirmation.

**Figure 13:**
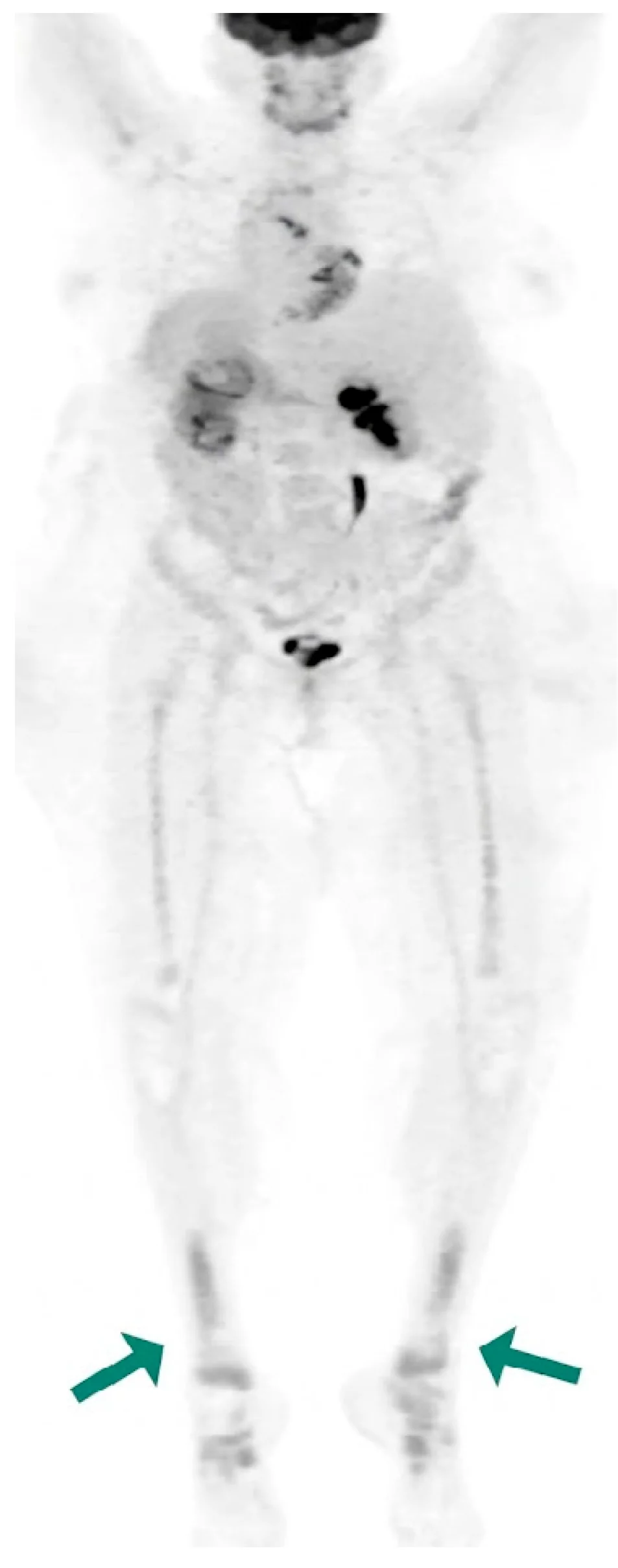
Whole-body ^18^F-FDG PET MIP demonstrates the systemic distribution of metabolically active disease, including bilateral and symmetric increased tracer uptake involving the distal lower extremities. Physiologic tracer uptake is visible in the brain, urinary tract, and urinary bladder. The whole-body projection provides an overview of both skeletal and extra-skeletal disease burden.

**Table 1:** Key objectives of multimodality imaging in the management of Erdheim–Chester disease (ECD).

**Diagnostic Triage**	Identification of pathognomonic signs (e.g., symmetric osteosclerosis, hairy kidneys, coated aorta)
**Biopsy Guidance**	Targeted sampling of highly avid/accessible extra skeletal sites (PET/CT guided)
**Staging and Mapping**	Full-body extent evaluation (CNS, Cardiac, Retroperitoneum, Musculoskeletal)
**Response Assessment**	Quantitative PERCIST metabolic criteria monitoring during targeted BRAF/MEK therapy

**Table 2:** Typical multimodality imaging findings of Erdheim–Chester disease according to organ system, with the principal role of each imaging modality and major differential diagnoses.

Organ/ System	Typical Imaging Manifestations	Key Imaging Modality/Role	Nuclear Imaging	Major Imaging Differentials
Skeleton	Bilateral symmetric metadiaphyseal osteosclerosis, cortical/endosteal thickening and periostitis, predominantly around knees/ankles	Radiography/CT for sclerosis; MRI for marrow infiltration	WBS: characteristic symmetric long-bone uptake; FDG-PET/CT for activity and systemic burden	LCH, metabolic/sclerosing bone disorders
Bone marrow	Marrow replacement/infiltration	MRI for marrow signal abnormalities	FDG-PET/CT may demonstrate metabolically active marrow disease	Myeloproliferative/myelodysplastic neoplasms
Cardiovascular	Coated aorta, pericardial thickening/effusion, right atrial pseudotumor, AV-groove and coronary encasement	Contrast CT for vascular anatomy; cardiac MRI for myocardial infiltration, edema and fibrosis	FDG-PET/CT for inflammatory/metabolic activity	Large-vessel vasculitis, IgG4-RD, lymphoma
Retroperitoneum/renal	Hairy kidney, perirenal rind, periaortic infiltration, proximal ureteral encasement, hydronephrosis	Contrast-enhanced CT; MRI for tissue characterization/fibrosis	FDG-PET/CT distinguishes metabolically active infiltration from predominantly fibrotic tissue	IgG4-RD, idiopathic retroperitoneal fibrosis, lymphoma
CNS/orbits	Pituitary stalk thickening, loss of posterior pituitary T1 bright spot, pachymeningeal masses, dentate/MCP abnormalities, retro-orbital infiltration	Contrast-enhanced brain/orbital MRI	PET has adjunctive value but is limited intracranially by physiological cerebral uptake	LCH, RDD, IgG4-RD, lymphoma, meningioma, CLIPPERS
Lung/pleura	Smooth interlobular and peribronchovascular septal thickening, ground-glass change, pleural thickening/effusion	HRCT	FDG-PET/CT for systemic/metabolic assessment	Pulmonary edema, infection, ILD, sarcoidosis
Skin	Xanthelasma-like and papulonodular lesions	Primarily clinical/dermatologic evaluation	Selected lesions may be FDG-avid in systemic assessment	Conventional xanthelasma, other histiocytoses

## Data Availability

No new data were created or analyzed in this study. Data sharing is not applicable to this article.

## Abbreviations

The following abbreviations are used in this manuscript:

ECD	Erdheim–Chester Disease
LCH	Langerhans Cell Histiocytosis
CT	Computed Tomography
HRCT	High-resolution Computed Tomography
MRI	Magnetic Resonance Imaging
WB-MRI	Whole Body Magnetic Resonance Imaging
PET	Positron Emission Tomography
PERCIST	PET Response Criteria in Solid Tumors
SUV	Standardized Uptake Value
HU	Hounsfield Unit
FLAIR	Fluid Attenuation Inversion Recovery
STIR	Short Tau Inversion Recovery
DWI	Diffusion Weighted Imaging
ADC	Apparent Diffusion Coefficient
LGE	Late Gadolinium Enhancement
BTFE	Balanced Turbo Field Echo
PSIR	Phase Sensitive Inversion Recovery
TSE	Turbo Spin Echo
CNS	Central Nervous System
AV	Atrioventricular
RCA	Right Coronary Artery
IAS	Interatrial Septum
SVC	Superior Vena Cava
IVC	Inferior Vena Cava
LVV	Large Vessel Vasculitis
IgG4-RD	IgG4-Related Disease
CLIPPERS	Chronic Lymphocytic Inflammation with Pontine Perivascular Enhancement Responsive to Steroids
